# The *PSE1* gene modulates lead tolerance in Arabidopsis

**DOI:** 10.1093/jxb/erw251

**Published:** 2016-06-21

**Authors:** Tingting Fan, Libo Yang, Xi Wu, Jiaojiao Ni, Haikun Jiang, Qi’an Zhang, Ling Fang, Yibao Sheng, Yongbing Ren, Shuqing Cao

**Affiliations:** ^1^School of Biotechnology and Food Engineering, Hefei University of Technology, Hefei, Anhui 230009, People’s Republic of China; ^2^Horticulture Institute, Anhui Academy of Agricultural Sciences, Hefei, Anhui 230031, People’s Republic of China

**Keywords:** Arabidopsis, glutathione, lead tolerance, phytochelatin, *PSE1*, *PDR12.*

## Abstract

Lead (Pb) is a dangerous heavy metal contaminant with high toxicity to plants. However, the regulatory mechanism of plant Pb tolerance is poorly understood. Here, we showed that the *PSE1* gene confers Pb tolerance in Arabidopsis. A novel Pb-sensitive mutant *pse1-1* (*Pb-sensitive1*) was isolated by screening T-DNA insertion mutants. *PSE1* encodes an unknown protein with an NC domain and was localized in the cytoplasm. *PSE1* was induced by Pb stress, and the *pse1-1* loss-of-function mutant showed enhanced Pb sensitivity; overexpression of *PSE1* resulted in increased Pb tolerance. *PSE1*-overexpressing plants showed increased Pb accumulation, which was accompanied by the activation of phytochelatin (PC) synthesis and related gene expression. In contrast, the *pse1-1* mutant showed reduced Pb accumulation, which was associated with decreased PC synthesis and related gene expression. In addition, the expression of *PDR12* was also increased in *PSE1*-overexpressing plants subjected to Pb stress. Our results suggest that *PSE1* regulates Pb tolerance mainly through glutathione-dependent PC synthesis by activating the expression of the genes involved in PC synthesis and at least partially through activating the expression of the ABC transporter *PDR12*/ABCG40.

## Highlight

*PSE1* regulates Pb tolerance mainly through GSH-dependent phytochelatin synthesis by activating the expression of the genes involved in phytochelatin synthesis and at least partially through activating the expression of *PDR12*.

## Introduction

Because of human activities and industrial processes, toxic heavy metals have been released or leached into the environment, which causes adverse effects on ecosystems (Ross, 1994; Steinnes, 2013). Among the heavy metals, lead (Pb) is a dangerous heavy metal contaminant that results in inhibition of metabolic processes, plant growth, and productivity (Meagher, 2000; Hall, 2002; Sharma and Dubey, 2005; Israr *et al.*, 2011; Ali *et al.*, 2014; Yang *et al.*, 2015). At the cellular and molecular level, Pb toxicity leads to disturbed water balance, mineral nutrition, and membrane organization; it also inhibits photosynthetic and enzyme activity, and reduces cell division (Uveges *et al.*, 2002; Israr *et al.*, 2011; Pourrut *et al.*, 2011). Furthermore, Pb causes many serious health problems in growing children and adults through food chain transfer (Kim *et al.*, 1996; Dudka and Miller, 1999; Duruibe *et al.*, 2007).

In order to protect against Pb stress, plants have developed a system of tolerance strategies, which include pumping out Pb at the plasma membrane; reducing the uptake of Pb; chelating or binding Pb to various thiol compounds in the cytosol, such as glutathione (GSH), phytochelatins (PCs), and metallothioneins (MTs), and then sequestering these into inactive organelles; and detoxifying Pb-induced reactive oxygen species (ROS) (Clemens, 2001; Hall, 2002; Verma and Dubey, 2003; Kim *et al.*, 2006; Zafari *et al.*, 2016).

One such strategy involves the induction of speciﬁc low molecular weight chelators, such as GSH and PCs, to bind and sequester Pb (Mehra *et al.*, 1995). GSH, a γ-Glu-Cys-Gly tripeptide, can act as a metal chelator through its thiol groups, as a cellular antioxidant, and as a ROS signaling molecule (Jozefczak *et al.*, 2012; Lin and Aaers, 2012; Seth *et al.*, 2012). Therefore, GSH plays an important role in the detoxification of heavy metals and in the metal-induced oxidative stress response. Many studies have confirmed that reduced GSH is the precursor of PCs, which are enzymatically synthesized oligomers containing (γ-Glu-Cys)_n_-Gly (*n*=2–11) (Rauser, 1990, 1995, 1999; Zenk, 1996; Cobbett, 2000). PCs sequester Pb to form a complex that is then transported into the vacuole by ATP-binding cassette (ABC) transporters, such as ABCC1 and ABCC2 (Cobbett and Goldsbrough, 2002; Clemens, 2006; Song *et al.*, 2010; Park *et al.*, 2012). Some transporters (such as ABCC1 and ABCC2) sequester heavy metals into inactive organelles (such as the vacuole), and other transporters have also been shown to extrude them across the plasma membrane, such as PDR12 (an ABC transporter, also named ABCG40) (Lee *et al.*, 2005; Verrier *et al.*, 2008).

Phytoremediation is one of the important ways to decontaminate Pb pollution (Suresh and Ravishankar, 2004; Pilon-Smits, 2005; Aken, 2008; M. Chen *et al.*, 2015). The identiﬁcation of genes involved in Pb accumulation and tolerance is the ﬁrst step towards their application in phytoremediation. It has been shown that some genes confer Pb tolerance, such as *EIN2*, *ABCP1*, *ATM3*, *PDR12*, and *SbLRR2* (Lee *et al.*, 2005; Kim *et al.*, 2006; Xiao *et al.*, 2008; Cao *et al.*, 2009; Zhu *et al.*, 2013). However, many novel genes involved in Pb tolerance require further identification. In this study, we identified a Pb-sensitive mutant, *pse1-1* (*Pb-sensitive1*), from the Arabidopsis Biological Resource Center (ABRC) stocks. *PSE1* encoded an unknown protein with an NC domain and was localized in the cytoplasm. We demonstrated that the transcription of the *PSE1* gene was induced by Pb stress, and overexpression of the *PSE1* gene led to increased Pb tolerance. The *PSE1* gene triggered GSH-dependent PC synthesis by activating the expression of the genes involved in the PC synthesis pathway, which resulted in enhanced Pb accumulation and tolerance. In addition, the expression of *PDR12* was increased in *PSE1*-overexpressing plants subjected to Pb stress, suggesting that a PDR12-dependent mechanism was partially involved in *PSE1*-mediated Pb tolerance.

## Materials and methods

### Plant materials, growth conditions, and treatments

*Arabidopsis thaliana* seeds of wild-type (WT; Col-0), *pse1-1* (SALK_046412; Alonso *et al.*, 2003), and transgenic plants were surface-sterilized and then germinated on half-strength Murashige and Skoog (1/2 MS) medium containing 1% (w/v) agar and 2% (w/v) sucrose at pH 5.8. The seeds were vernalized at 4 °C for 3 d in the dark. The plants were grown in a controlled culture room at 22 °C under long-day (16h of light/8h of dark) conditions with a light intensity of 100 µmol m^−2^ s^−1^.

For the Pb tolerance test, seeds of the WT, the *pse1-1* mutant, and the transgenic plants were germinated and grown vertically on 1/2 MS medium in the absence or presence of the indicated concentrations of Pb(NO_3_)_2_ or the glutathione synthesis inhibitor, buthioninesulphoximine (BSO; Sigma) for 2 weeks, to check whether Pb tolerance in *PSE1*-overexpressing plants is glutathione dependent. Then, the plants were weighed and their root lengths were measured. There were triplicate replicates for the Pb tolerance test, and ~30 plants were used for each measurement. For Pb-inducible gene expression analysis, the plants were grown for 2 weeks on 1/2 MS medium and then exposed to a solution of 0.5mM Pb(NO_3_)_2_ or water (control) at the designated time points specified in the text.

### Generation of PSE1-overexpressing plants and PSE1- complemented plants

To generate *PSE1*-overexpressing plants, the coding region of *PSE1* (At5g06370) was amplified by PCR using the specific primers PSE1-FP-XhoI and PSE1-RP-HindIII (see Supplementary Table S1 at *JXB* online), followed by cloning into the pCAMBIA1301 vector at the *Xho*I and *Hin*dIII restriction sites. To generate *PSE1*-complemented plants, the promoter of *PSE1* was amplified by the primers PSE1_pro_-FP-KpnI and PSE1_pro_-RP-XhoI (Supplementary Table S1) and then cloned into *pXB93* (a *pART27* derivative including *MCS-GUS*, kindly supplied by X.B. Peng and M.X. Sun) using the *Kpn*I and *Xho*I restriction sites. Then, the amplified fragment of PSE1 cDNA was cloned into the above construct at the *Xho*I and *Hin*dIII restriction sites. The *35S::PSE1* and *PSE1*_*pro*_ *::PSE1* constructs were introduced into the *Agrobacterium* GV3101 strain, which was then used to transform the WT or the *pse1-1* mutant using the floral dip method (Clough *et al.*, 1998). All transgenic lines used in this study were T_3_ homozygous plants with single-copy insertions, from which two lines (OE-1 and OE-2; COM1 and COM2) were selected for further analysis.

### RNA extraction, and quantitative RT-PCR (qRT-PCR) analysis

Total seedling RNA was extracted using Trizol Reagent (Invitrogen, CA) and used in a reverse transcription reaction using PrimeScript™ Reverse Transcriptase (TAKARA, Japan) and the oligo(dT)_15_ primer (TAKARA, Japan) following the manufacturer’s instructions. For qRT-PCR analysis, the reaction was performed according to the instructions provided for the Bio-Rad iCycleriQ system (Bio-Rad Laboratories, CA, USA) and platinum SYBR Green qPCR SuperMix-UDG (Invitrogen, CA, USA). All experiments were performed at least in triplicate, and the *Actin11* (At3g12110) gene was used as an internal control. The primers used are listed in Supplementary Table S1.

### GUS (β-glucuronidase) staining and activity assay

In order to generate *PSE1*_*pro*_ *::GUS* transgenic plants, a fragment of the *PSE1* promoter was amplified by PCR using the primers PSE1_pro_-FP-KpnI and PSE1_pro_-RP-XhoI (Supplementary Table S1). The PCR product was cloned into *pXB93* using the *Kpn*I and *Xho*I restriction sites. The construct was transferred into the WT plants using the *Agrobacterium* strain *GV3101* (Clough *et al.*, 1998). Homozygous transgenic plants were germinated on 1/2 MS medium for 2 weeks or grown in a greenhouse for 4–6 weeks. All samples were collected and stained with 5-bromo4-chloro-3-indoyl-β-d-glucuronide (X-Gluc) as reported previously (Fan *et al.*, 2013).

### Subcellular localization of PSE1

To build up the construct of 35S::PSE1-GFP, the PSE1 coding region was obtained by PCR using specific primers and then cloned as a *Kpn*I/*Xho*I fragment into pXB94 (a pART27 derivative including 35S:MCS-eGFP, kindly supplied by X.B. Peng and M.X. Sun). The construct was transferred into the *Agrobacterium* strain GV3101 by electrophoresis, and the WT plants were transformed. The transgenic lines were used for subcellular localization analysis. The subcellular localization of PSE1 was visualized using confocal microscopy (Leica TCS-SP8; Wetzlar, Hesse-Darmstadt, Germany).

### Measurement of Pb content

The WT, *PSE1-OE*, and *pse1-1* mutant plants were grown on 1/2 MS medium in the absence or presence of 0.5mM Pb(NO_3_)_2_ for 2 weeks and then sampled to determine the Pb content, which was analysed using the method described by Kim *et al.* (2006). Digested samples were analysed using an atomic absorption spectrometer (Solaar M6, Thermo Fisher, Waltham, MA, USA). A standard reference material was not used to assess the efficiency of the acid digestion procedure; therefore, while the relative concentrations of Pb in WT, *PSE1-OE*, and *pse1-1* mutant plants are reliable, the actual concentrations reported may be less than the true values.

### Determination of GSH and PC contents

The WT, *PSE1-OE*, and *pse1-1* mutant plants were grown on 1/2 MS medium for 2 weeks and treated or not treated with 0.5mM Pb(NO_3_)_2_ for 12h, after which they were sampled to determine the GSH and PC contents. The method used to determine the GSH/PC contents followed J. Chen *et al.* (2015).

### Accession numbers

Sequence data from this article can be found in the Arabidopsis Genome Initiative or GeneBank/EMBL database under the following accession numbers: *PSE1* (At5g06370), *GSH1* (At4g23100), *GSH2* (At5g27380), *PCS1* (At5g44070), *PCS2* (At1g03980), *GR1* (At3g24170), *GR2* (At3g54660), *ABCC1* (At1g30400), *ABCC2* (At2g34660), *PDR8* (At1g59870), *ATM3* (At5g58270), *PDR12* (At1g15520), *ACBP1* (At5g53470), and *ACTIN11* (At3g12110).

## Results

### The *pse1-1* mutant is Pb sensitive

**Fig. 1.**
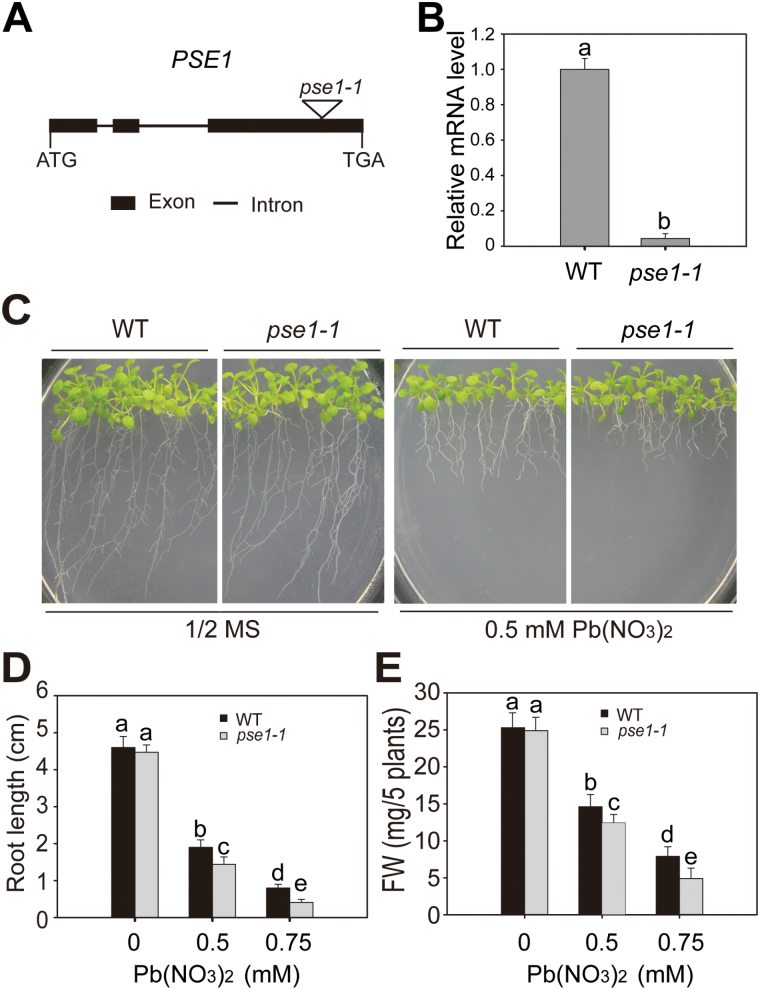
Pb sensitivity of the *pse1-1* mutant. (A) A schematic of the genomic region flanking the T-DNA insertion site in the *pse1-1* mutant. Black boxes and black lines indicate exons and introns, respectively, in *PSE1*. The triangle indicates the site of T-DNA insertion. (B) qRT-PCR analysis of *PSE1* transcription levels in 2-week-old seedlings of the *pse1-1* mutant and wild-type (WT) plants. *ACTIN11* was used as the internal control. Data are presented as the means SE of three replicate experiments. Statistical significance was determined by Student’s *t*-test; a significant difference (*P*<0.05) is indicated by different lower case letters. (C) Phenotypes of the *pse1-1* mutant under Pb stress. Seedlings were grown on 1/2 MS medium with or without 0.5mM Pb(NO_3_)_2_ for 2 weeks. (D and E) Root length (D) and fresh weight (E) of the WT and *pse1-1* grown on 1/2 MS medium with or without 0.5/0.75mM Pb(NO_3_)_2_ for 2 weeks. Three independent experiments were performed with similar results, each with three biological replicates. Five plants per genotype from one plate were measured for each replicate. Data are presented as the means ±SE, *n*=3. Statistical significance was determined using ANOVA in combination with post-hoc tests (Tukey); significant differences between the WT and *pse1-1* (*P*<0.05) are indicated by different lower case letters. (This figure is available in colour at *JXB* online.)

To identify novel genes involved in regulating the response to Pb stress in Arabidopsis, the mutants from the ABRC stock were screened. One T-DNA insertion mutant, designated *pse1-1* (*Pb-sensitive1*; *SALK_046412*; Alonso *et al.*, 2003), was more sensitive to Pb stress than the WT (Fig. 1). Using PCR analysis, we found that the T-DNA of the *pse1-1* mutant was inserted in At5g06370. The At5g06370 gene is a single-copy gene possessing three exons and two introns, and is located on chromosome 5 (Fig. 1A). The transcript expression of *PSE1* in *pse1-1* was significantly reduced according to the qRT-PCR results (*P*<0.05; Fig. 1B). There were no signiﬁcant differences in growth between the WT and *pse1-1* plants grown on 1/2 MS medium. However, when 0.5mM Pb(NO_3_)_2_ was added, the *pse1-1* mutant was more sensitive to Pb stress than the WT (Fig. 1C). The root length and fresh weight of the *pse1-1* mutant were significantly inhibited compared with the WT plants in a Pb concentration-dependent manner (*P*<0.05; Fig. 1D, E). We further generated genetically complemented *pse1-1* mutant plants using the full-length *PSE1* under the *PSE1* promoter. The transcript levels of *PSE1* were similar or slightly higher in the transgenic than in the WT plants (*P*>0.05; Supplementary Fig. S1A). The complemented lines (COM1 and COM2) reversed the Pb-sensitive phenotype of the *pse1-1* mutant to the level of the WT (Supplementary Fig. S1B, C).Together, these results indicate that loss-of-function of *PSE1* is responsible for the Pb-sensitive phenotype.

To test whether *pse1-1* was also tolerant to other stresses, the *pse1-1* mutant was grown on 1/2 MS medium containing CdCl_2_, Na_3_AsO_4_, ZnSO_4_, and H_2_O_2_. Upon treatment with CdCl_2_, Na_3_AsO_4_, and ZnSO_4_, no signiﬁcant difference was observed between the *pse1-1* mutant and the WT plants (*P*>0.05; Supplementary Fig. S2A–C). However, the *pse1-1* mutant was more sensitive to H_2_O_2_ than the WT (*P*<0.05; Supplementary Fig. S2D). In this study, we focused on the *PSE1*-mediated Pb tolerance mechanism.

### Overexpression of *PSE1* results in increased Pb tolerance

**Fig. 2.**
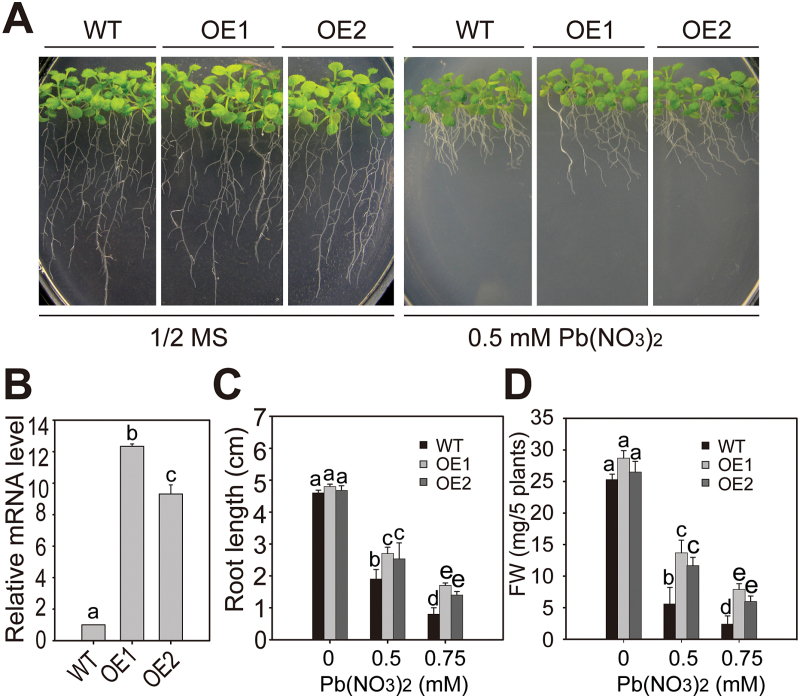
Pb tolerance in the *PSE1*-overexpressing lines. (A) Growth of wild-type (WT) and *PSE1*-overexpressing lines (OE1 and OE2) under Pb stress. Seedlings were grown on 1/2 MS medium with or without 0.5mM Pb(NO_3_)_2_ for 2 weeks. (B) qRT-PCR analysis of *PSE1* transcription levels in 2-week-old seedlings of the WT and *PSE1*-overexpressing lines (OE1 and OE2). *ACTIN11* was used as the internal control. Data are presented as the means ±SE of three replicate experiments. Statistical significance was determined by Student’s *t*-test; a significant difference (*P*<0.05) is indicated by different lower case letters. (C and D), Root length (C) and fresh weight (D) of the WT and *PSE1*-overexpressing lines (OE1 and OE2) grown on 1/2 MS medium with or without 0.5/0.75mM Pb(NO_3_)_2_ for 2 weeks. Three independent experiments were performed with similar results, each with three biological replicates. Five plants per genotype from one plate were measured for each replicate. Data are presented as the means ±SE, *n*=3. Statistical significance was determined using ANOVA in combination with post-hoc (Tukey) tests; significant differences between the WT and *PSE1*-overexpressing lines (*P*<0.05) are indicated by different lower case letters. (This figure is available in colour at *JXB* online.)

To determine further the role of *PSE1* in Pb tolerance, we generated *PSE1*-overexpressing transgenic plants. The full-length cDNA was cloned under the control of the *Cauliflower mosaic virus* (CaMV) 35S promoter. At least 10 transgenic lines were obtained, and two independent T_3_ homozygous lines (OE1 and OE2) were used for further analyses (Fig. 2). The transcript level of *PSE1* was higher in *PSE1*-overexpressing transgenic plants than in the WT plants (*P*<0.05; Fig. 2A). In 1/2 MS medium, the growth of the WT and *PSE1*-overexpressing plants was not different; however, in 1/2 MS medium containing Pb(NO_3_)_2_, the *PSE1*-overexpressing plants were more tolerant than the WT plants (*P*<0.05; Fig. 2B–D). Under normal growth conditions, the *pse1-1* mutant, *PSE1*-overexpressing plants, and the WT exhibited similar growth and development in both vegetative and reproductive phases (Supplementary Fig. S3). Together, these results suggest that *PSE1* plays an important role in regulating Pb tolerance.

### The *PSE1* gene is induced by Pb stress

**Fig. 3.**
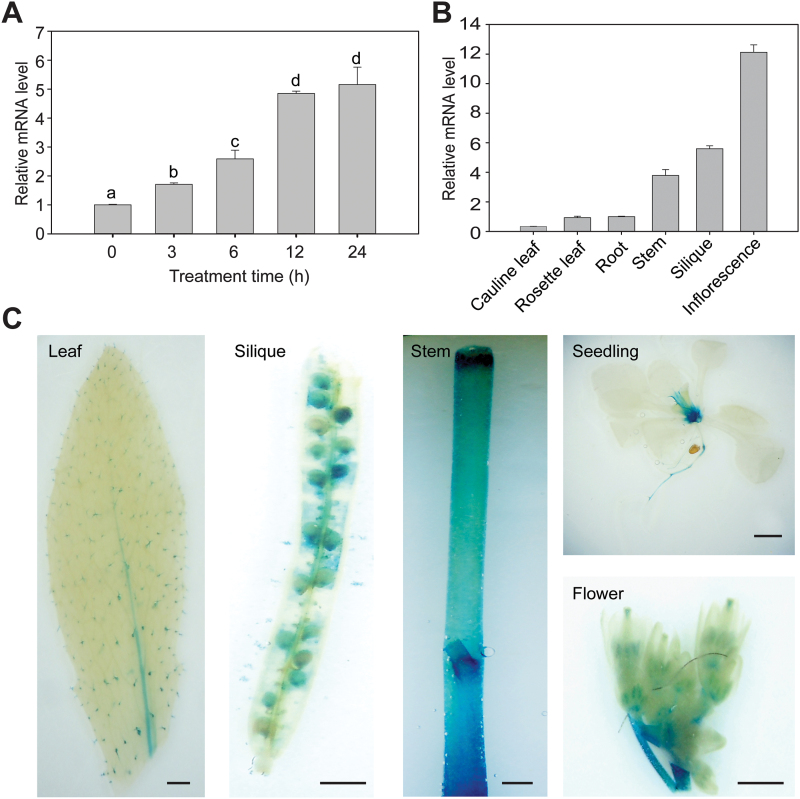
Expression pattern of the *PSE1* gene. (A) Expression of *PSE1* in response to Pb stress. Two-week-old wild-type (WT) seedlings grown on 1/2 MS medium were treated with Pb(NO_3_)_2_ (0.5mM) for 0, 3, 6, 12, and 24h, and then the tissues were harvested for qRT-PCR analysis. *ACTIN11* was used as the internal control. Data are presented as the means ±SE of three replicate experiments. Statistical significance was determined using ANOVA in combination with post-hoc (Tukey) tests; significant differences between treated and control plants (*P*<0.05) are indicated by different lower case letters. (B) qRT-PCR analysis of *PSE1* transcription in different tissues of WT plants. mRNAs were isolated from cauline leaves, rosette leaf, root, stem, silique, and inflorescences of 6-week-old WT plants. *ACTIN11* was used as the internal control. (C) Histochemical analysis of *PSE1*_*Pro*_ *::GUS* transgenic lines. Leaf (4-week-old), silique (6-week-old), stem (6-week-old), seedling (2-week-old), and flower (6-week-old) samples after 6h of GUS staining are shown in the panels from left to right. Scale bar=1mm. (This figure is available in colour at *JXB* online.)

To examine whether the expression of *PSE1* is induced by Pb stress, the WT seedlings were treated at different time points after Pb treatment. As shown in Fig. 3A, the expression of *PSE1* was significantly induced by Pb stress (*P*<0.05). To test the expression profile of *PSE1* in various tissues, the transcript levels of *PSE1* were quantified using qRT-PCR. *PSE1* was found to be expressed in most of the examined tissues and highly expressed in the inflorescence, silique, and stem (Fig. 3B). To explore further the spatial expression pattern, we generated transgenic Arabidopsis seedlings expressing a *PSE1*_*pro*_ *::GUS* transgene. Histochemical analyses showed that GUS activity was detected in most organs, especially in the inflorescence, silique, and stem (Fig. 3C), which is consistent with the qRT-PCR results shown in Fig. 3B.

### PSE1 is localized in the cytoplasm

**Fig. 4.**
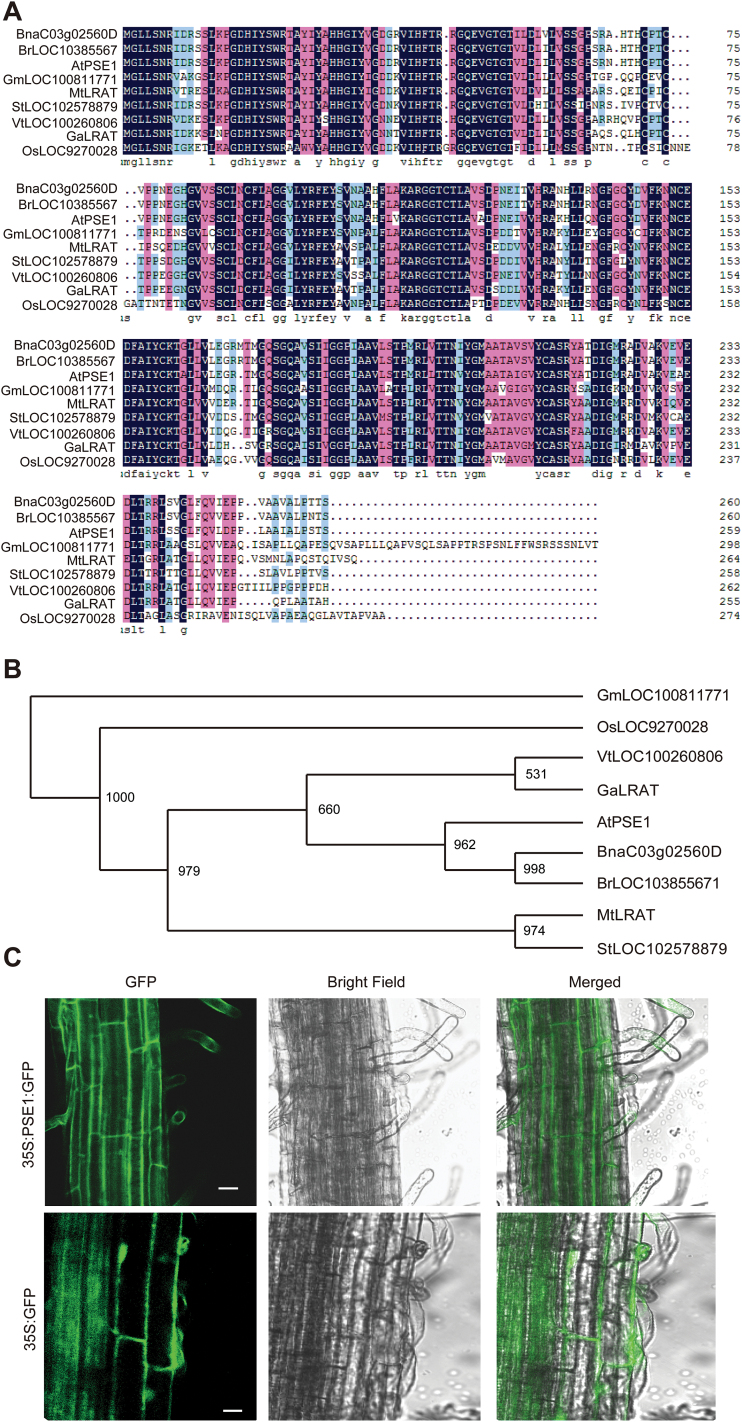
Subcellular localization of PSE1. (A) Multiple sequence alignment of PSE1 and several homologues from other species through DNAMAN software. (B) Phylogenic tree of PSE1 and homologues using Clustalx in combination with Phylip. (C) Subcellular localization of the 35S::PSE1–GFP fusion protein in transgenic lines. The expression of 35S::GFP was used as a control. Scale bar=20 μm. (This figure is available in colour at *JXB* online.)

The *PSE1* gene contains three exons and two introns, and encodes a 259 amino acid protein with one predicted NC domain (IPR0077053; Tsai *et al.*, 2009). BLAST analysis of PSE1 in the non-redundant database of NCBI revealed that it shares >70% sequence similarity with other proteins such as BnaC03g02560D (ID:CDX81002, *Brassica napus*), BrLOC103855671 (ID:XP_009130938, *Brassica rapa*), VtLOC100260806 (ID:XP_002266364, *Vitis vinifera*), OsLOC9270028 (ID:XP_015640129, *Oryza sativa* Japonica Group), GmLOC100811771 (ID: XP_014623907, *Glycine max*), GaLRAT (ID:KHG06143, *Gossypium arboreum*), MtLRAT(ID:XP_013467890, *Medicago truncatula*), and StLOC102578879 (ID: XP_006348469, *Solanum tuberosum*) (Fig. 4A, B).

PSE1 is predicted by the SubCellular Proteomic Database (SUBA3) (http://suba.plantenergy.uwa.edu.au/flatfile.php?id=AT5G06370.1) to be localized in the cytoplasm. To test this prediction, we fused the coding region of PSE1 at the N-terminus of a green ﬂuorescent protein (GFP) fragment, and the fusion construct was transformed into WT *A. thaliana*. The fluorescent signal was clearly visible in the cytoplasm (Fig. 4C). Thus, our data demonstrate that PSE1 is indeed localized in the cytosplasm.

### PSE1-mediated Pb tolerance is GSH dependent

**Fig. 5.**
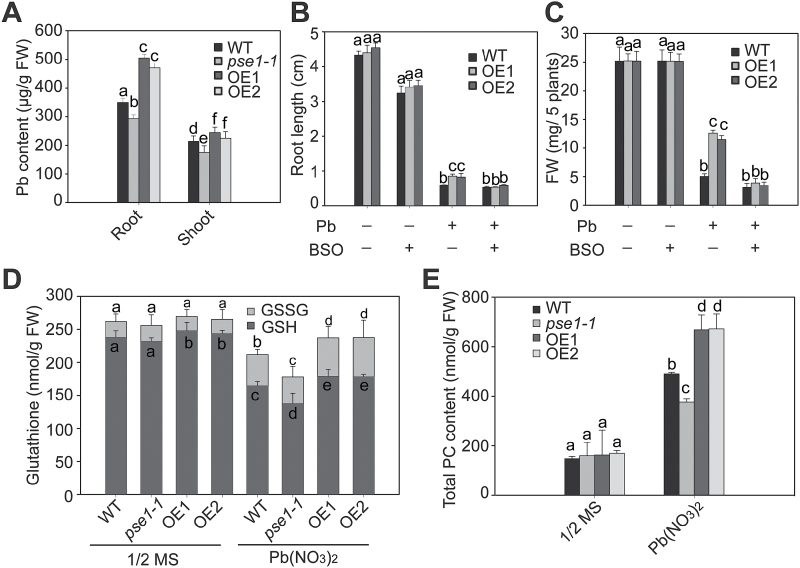
*PSE1* regulates Pb tolerance through the GSH-dependent pathway. (A) Measurements of Pb contents in wild-type (WT), *pse1-1* mutant, and *PSE1*-OE lines. These plants were grown on 1/2 MS medium with 0.5mM Pb(NO_3_)_2_ for 2 weeks, and roots and shoots of these samples were collected for Pb content measurements. (B and C) Effect of BSO on root length (B) and fresh weight (C) of WT and *PSE1*-OE plants. Seedlings were grown on 1/2 MS medium with (+) or without (–) 0.5mM Pb(NO_3_)_2_ or 0.1mM BSO for 2 weeks, and then their root lengths and fresh weights were measured. Three independent experiments were performed with similar results, each with three biological replicates. Five plants per genotype from one plate were measured for each replicate. Data are presented as the means ±SE, *n*=3. (D and E) Glutathione (D) and PC (E) contents in the *pse1-1* mutant and the *PSE1*-OE line. Seedlings were grown on 1/2 MS medium for 2 weeks and then treated or not with 0.5mM Pb(NO_3_)_2_ for 12h, and their GSH/GSSG (D) and PC (E) contents were quantified. Data are presented as the means ±SE of three replicate experiments. Statistical significance was determined using ANOVA in combination with post-hoc (Tukey) tests; significant differences (*P*<0.05) between parameters are indicated by different lower case letters.

To investigate whether the *PSE1*-mediated Pb tolerance is associated with Pb accumulation, we analysed the Pb content in the WT, *pse1-1* mutant, and *PSE1*-overexpressing plants that were treated with Pb(NO_3_)_2_. We found that the Pb contents were significantly increased in the *PSE1*-overexpressing plants and significantly decreased in the *pse1-1* mutant in comparison with the WT control (*P*<0.05; Fig. 5A). Thus, a sequestration mechanism might be involved in *PSE1*-mediated Pb tolerance.

GSH is important for heavy metal detoxification via vacuolar sequestration (Lin *et al.*, 2012; J. Chen *et al.*, 2015), and it can bind Pb(II) ions as a precursor of phytochelation (Grill *et al.*, 1989; Cobbett and Goldsbrough, 2002; Noctor *et al.*, 2012). To test whether *PSE1*-mediated Pb tolerance is related to GSH, a GSH synthesis inhibitor, BSO, was used to treat the plants. WT and *PSE1*-overexpressing plants did not show significant differences in growth on medium containing BSO alone; however, the root length and fresh weight were higher in *PSE1*-overexpressing plants than in the WT plants in the medium containing Pb(NO_3_)_2_ (Fig. 5B, C). When BSO was added together with Pb(NO_3_)_2_, the phenotype of elongated root and increased fresh weight in the *PSE1*-overexpressing plants disappeared (Fig. 5B, C). These results suggest that the mechanism of *PSE1*-mediated Pb tolerance is GSH dependent.

To confirm further the role of GSH in *PSE1*-mediated Pb tolerance, we measured the concentrations of GSH in the WT, *pse1-1* mutant, and *PSE1*-overexpressing plants in response to Pb stress. Without Pb treatment, no significant difference was detected in total glutathione (*P*>0.05, GSH plus 2GSSG) among the WT, *PSE1*-overexpressing plants, and the *pse1-1* mutant; however, the *PSE1*-overexpressing plants contained a more reduced form of GSH whereas the *pse1-1* mutant possessed less GSH in comparison with the WT (Fig. 5D). When challenged by Pb stress, the GSH concentrations decreased signiﬁcantly in the *PSE1*-overexpressing plants, the *pse1-1* mutant, and the WT; however, compared with the WT plants, a slight decrease was observed in the *PSE1*-overexpressing plants while a higher decrease existed in the *pse1-1* mutant (*P*<0.05; Fig. 5D).

The total PC content was also analysed in the *PSE1*-overexpressing plants, *pse1-1* mutant, and WT after treatment with Pb stress. Compared with the WT, the total PC content was significantly increased in the *PSE1*-overexpressing plants but decreased in the *pse1-1* mutant in the presence of Pb (*P*<0.05; Fig. 5E). These results suggest that *PSE1*-mediated Pb accumulation and tolerance is associated with increased PC synthesis.

### *PSE1* positively regulates Pb tolerance by activating the expression of PC synthesis-related genes

**Fig. 6.**
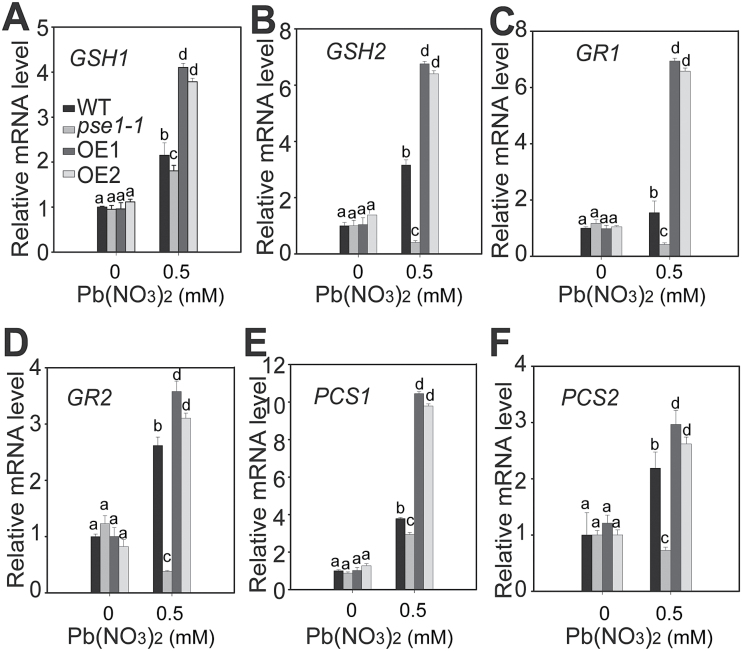
qRT-PCR analysis of the genes involved in PC synthesis. Quantitative analysis of the transcription of genes involved in GSH/PC synthesis in the *pse1-1* mutant and *PSE1*-OE lines, including *GSH1* (A), *GSH2* (B), *GR1* (C), *GR2* (D), *PCS1* (E), and *PCS2* (F). The wild type (WT), the *pse1-1* mutant and *PSE1*-OE lines were grown on 1/2 MS medium for 2 weeks and treated or not with 0.5mM Pb(NO_3_)_2_ for 12h, after which their mRNAs were isolated for qRT-PCR analysis. *ACTIN11* was used as the internal control. Data are presented as the means ±SE of three replicate experiments.. Statistical significance was determined using ANOVA in combination with post-hoc (Tukey) tests; significant differences (*P*<0.05) between the *pse1-1*/*PSE1-OE* line and the WT are indicated by different lower case letters.

The above results suggest that *PSE1* regulates Pb tolerance through the GSH-dependent PC synthesis pathway (Xiang and Oliver, 1998; Zhu *et al.*, 1999*a*, *b*; Kim *et al.*, 2006; Verbruggen *et al.*, 2009*a*, *b*; Noctor *et al.*, 2012; Seth *et al.*, 2012). Therefore, we analysed the genes involved in this pathway. Under Pb stress, higher transcription levels of a few key genes were detected, including *GSH1*, *GSH2*, *GR1*, *GR2*, *PCS1*, and *PCS2*, in the *PSE1*-overexpressing plants than in the WT plants; in contrast, their transcription levels were signiﬁcantly decreased in the *pse1-1* mutant in comparison with the WT control (*P*<0.05; Fig. 6). In addition, the transcription levels of other genes involved in regulating Pb tolerance, including *PDR12*, *ATM3*, *ACBP1*, *ABCC1*, *ABCC2*, and *PDR8* (Lee *et al.*, 2005; Kim *et al.*, 2006, 2007; Xiao *et al.*, 2008; Park *et al.*, 2012), were analysed. There were no significant differences in the genes *ATM3*, *ACBP1*, *ABCC1*, *ABCC2*, and *PDR8* among the WT, *PSE1*-overexpressing plants, and the *pse1-1* mutant (Supplementary Fig. S4). However, the induction of *PDR12* was significantly higher in the *PSE1*-overexpressing plants but lower in the *pse1-1* mutant (Supplementary Fig. S4). These results suggest that the GSH-dependent PC synthesis pathway is a major mechanism contributing to PSE1-mediated Pb tolerance, and PDR12-dependent Pb tolerance might be another complementary mechanism. Moreover, *PDR8* was also induced but had no significant difference among the WT, *PSE1*-overexpressing plants, and the *pse1-1* mutant. This may explain why there was no signiﬁcant difference in growth between the *pse1-1* mutant and the WT plants in the presence of Cd.

## Discussion

**Fig. 7.**
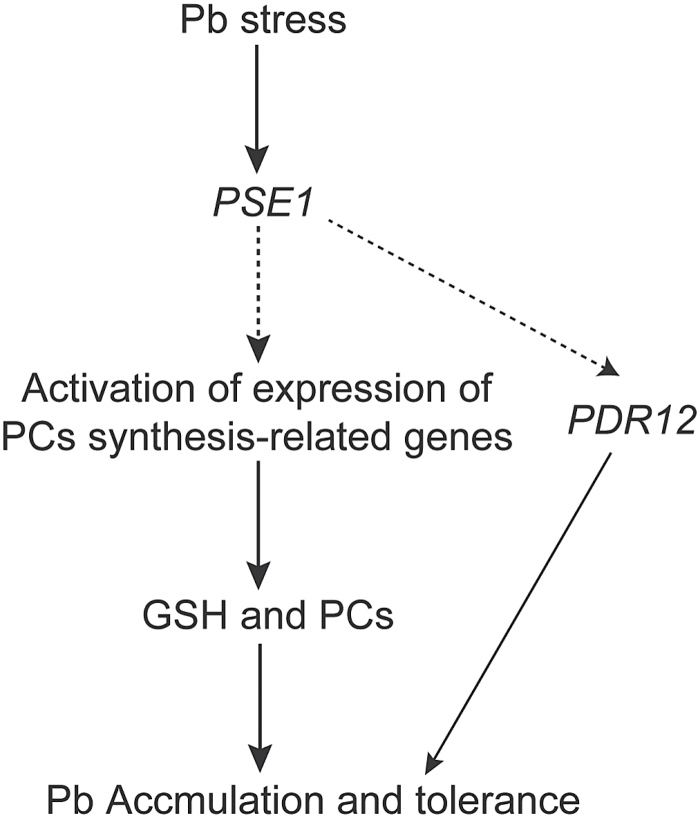
A working model for the role of PSE1 in regulating Pb accumulation and tolerance. Pb stress rapidly induced the expression of PSE1, which triggered GSH-dependent PC synthesis by activating the expression of the genes involved in the PC synthesis pathway. Moreover, the induced expression of *PSE1* also led to increased expression of the Pb pump *PDR12*. Together, these factors triggered Pb-activated PC synthesis and the pumping out of Pb, which resulted in enhanced Pb accumulation and tolerance. Solid lines indicate direct regulation between upstream and downstream factors, while dashed lines indicate indirect regulation between these factors.

In this study, we explored the role of *PSE1* in Pb tolerance in Arabidopsis by investigating the effects of gain and loss of function of this gene on plant responses to Pb stress. Our results demonstrated that *PSE1* contributed to Pb tolerance: Pb stress rapidly induced *PSE1*, which then triggered GSH-dependent PC synthesis and GSH-independent PDR12 transport through the co-ordinated regulation of the expression of PC synthesis-related genes and *PDR12*. This cascade of events resulted in enhanced Pb accumulation and tolerance (Fig. 7).

The molecular mechanisms for Pb tolerance are still not well understood in plants, and only a few genes have been identified and characterized (Clemens, 2001; Hall, 2002; Lee *et al.*, 2005; Kim *et al.*, 2006; Xiao *et al.*, 2008; Cao *et al.*, 2009). Here, we showed that *PSE1* encodes a member of the NC protein family that plays an important role in Pb tolerance. First, the root length and fresh weight of the *pse1-1* mutant grew less than those of the WT in the presence of Pb, while the complemented plants had a reversed Pb sensitivity phenotype (Fig. 1; Supplementary Fig. S1). Secondly, overexpression of *PSE1* led to improved Pb tolerance (Fig. 2). Thirdly, the expression of *PSE1* was induced by Pb (Fig. 3). Thus, *PSE1* is required for Pb tolerance in Arabidopsis.

A GSH-dependent PC pathway is one of the most important mechanisms contributing to Pb resistance (Mehra *et al.*, 1995). Many studies have shown that PCs play an important role in heavy metal detoxiﬁcation in living organisms (Verbruggen *et al.*, 2009*a*, *b*; Lin *et al.*, 2012). PCs are induced by a wide range of heavy metals (Grill *et al.*, 1987; Maitani *et al.*, 1996). In addition, they bind to Pb to form stable metal–PC complexes and are sequestered in the vacuole to inhibit Pb toxicity in the cell (Cobbett and Goldsbrough, 2002; Clemens, 2006; Song *et al.*, 2010; Park *et al.*, 2012). GSH, as the metabolic precursor of PCs, is involved in heavy metal detoxification in plants (Cobbett, 2002; Ball *et al.*, 2004). Overexpression of *GSH1*, *GSH2*, and *PCS1* involved in PC biosynthesis has been shown to increase Cd accumulation and tolerance (Zhu *et al.*, 1999*a*, *b*; Brunetti *et al.*, 2011). Our results demonstrated that *PSE1* enhanced Pb accumulation and tolerance though the induction of PC synthesis and the activation of the expression of genes involved in PC synthesis, including *GSH1*, *GSH2*, *PCS1*, *PCS2*, *GR1*, and *GR2* (Figs 5, 6). These results suggest that *PSE1* is an important regulator of the GSH-dependent PC synthesis pathway that contributes to Pb accumulation and tolerance. Many metal transporters have been identified as important components involved in heavy metal detoxiﬁcation in diverse organisms; these transporters remove heavy metals from the cytoplasm to avoid toxicity in cells. Some transporters extrude them across the plasma membrane and others sequester them into inactive organelles, such as the vacuole (Lee *et al.*, 2005). In Arabidopsis, AtPDR12, AtATM3, ABCC1, ABCC2, and AtPDR8 were reported to be associated with Pb tolerance (Cobbett and Goldsbrough, 2002; Lee *et al.*, 2005; Clemens, 2006; Kim *et al.*, 2006, 2007; Song *et al.*, 2010; Park *et al.*, 2012). AtPDR12 was identified as the first transporter gene that served specifically as an efﬂux pump for Pb exclusion, and the AtPDR12-dependent mechanism is a GSH-independent mechanism (Lee *et al.*, 2005). AtATM3 responded to both Pb and Cd stresses and may mediate GSH-conjugated Cd transport across the mitochondrial membrane (Kim *et al.*, 2006). AtPDR8 is induced by both Pb and Cd, and is involved as an efﬂux pump of Cd or Cd conjugates at the plasma membrane (Kim *et al.*, 2007). ABCC1 and ABCC2 transported a PC–Pb complex into the vacuole (Cobbett and Goldsbrough, 2002; Clemens, 2006; Song *et al.*, 2010; Park *et al.*, 2012). It is noteworthy that the expression of *PDR12* was induced in the *PSE1*-overexpressing plants (see Supplementary Fig. S4). However, as shown in Fig. 5A–C, the concentration of Pb in the *PSE1*-overexpressing plants was much higher than that in the WT, and the Pb tolerance phenotype of the *PSE1*-overexpressing plants almost disappeared in the presence of BSO. These results suggest that *PSE1*-mediated Pb tolerance may be mainly related to a GSH-dependent mechanism and, at least in part, depends on a PDR12-dependent detoxification mechanism. Based on the facts that the expression of genes involved in PC synthesis and *AtPDR12* was activated by *PSE1* and that PSE1 was localized in the cytoplasm, we speculated that PSE1 might interact with some special transcription factors or other signalling components to regulate the expression of these genes, thereby leading to the activation of the GSH-dependent PC synthesis and PDR12-dependent detoxification pathways. However, these interacting partners require further identification.

The multiple sequence alignment and phylogenic tree results showed that PSE1 belongs to the NC protein family along with LRAT, an expanding family of homologous proteins (Fig. 4). However, the biochemical function of PSE1 requires further study in future research. *Retinoid-inducible gene 1* (*RIG1*) has recently been classified into this family, and Tsai *et al.* (2009) reported that the NC domain, especially the NC motif, plays the major role in RIG1-mediated pro-apoptotic activity. The cysteine residues in the amino acid sequence of PSE1 suggest that PSE1 might be redox regulated, and the glutathione/glutaredoxin system has been reported to regulate the protein redox state (Cheng *et al.*, 2006; Zaffagnini *et al.*, 2012; Krasensky *et al.*, 2014). This indicates that PSE1 may play a role in the oxidative stress response. Interestingly, we found that the growth of the *pse1-1* mutant was not different from that of the WT plants under Cd, Zn, or As stress, but *pse1-1* was more sensitive to H_2_O_2_ than the WT plants. The possible mechanism of PSE1-mediated H_2_O_2_ tolerance may be related to ROS production or anti-oxidative capacity. However, further research is needed to ascertain a possible link between ROS and Pb tolerance conferred by *PSE1*.

In summary, our results suggest that the *PSE1* gene plays an important role in regulating Pb tolerance in Arabidopsis through the GSH-dependent PC synthesis and PDR12-dependent detoxification pathways.

## Supplementary data

Supplementary data are available at *JXB* online.

**Figure S1.** The *PSE1*-complemented plants exhibited a Pb-insensitive phenotype different from that of the *pse1-1* mutant.

**Figure S2.** Phenotype of wild-type (WT) and *pse1-1* mutant seedlings in response to Cd, As, Zn, or H_2_O_2_ stress.

**Figure S3.** Phenotypes of wild-type (WT) and *pse1-1* plants in soil-filled pots.

**Figure S4.** Transcript levels of Pb stress-related genes in WT, *pse1-1* mutant, and *PSE1*-overexpressing plants subjected to Pb stress.

**Table S1.** Primer sequences used for cloning and qRT-PCR.

Supplementary Data
