# Supplementary material for: The PSE1 gene modulates lead tolerance in Arabidopsis
Source: J Exp Bot. 2016 Jun 21;67(15):4685–95. doi: 10.1093/jxb/erw251 (PMC4973742; doi:10.1093/jxb/erw251)
Supplement: Supplementary Data [file supp_67_15_4685__index.html]

The PSE1 gene modulates lead tolerance in Arabidopsis — The PSE1 gene modulates lead tolerance in Arabidopsis — Supplementary Data 

# The *PSE1* gene modulates lead tolerance in Arabidopsis

## Supplementary Data

Data files

- supplementary\_figure\_S1\_S4\_Table\_S1.pdf - Supplementary Data
