# Supplementary material for: The PSE1 gene modulates lead tolerance in Arabidopsis
Source: J Exp Bot. 2016 Jun 21;67(15):4685–95. doi: 10.1093/jxb/erw251 (PMC4973742; doi:10.1093/jxb/erw251)
Supplement: Supplementary Data [file supp_erw251_supplementary_figure_S1_S4_Table_S1.pdf]

Supplemental Data

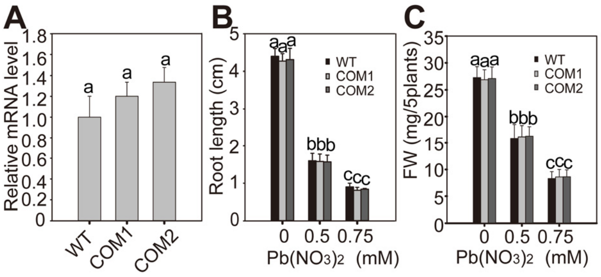

Figure S1 The *PSE1*-complemented plants exhibited a Pb-insensitive phenotype different to that of the *pse1-1* mutant. (A) Analysis of the *PSE1* transcript expression in the *PSE1*-complement plants using qRT-PCR. (B) and (C) Measurements of the root length (B) and fresh weight (C) of plants grown on 1/2 strength MS medium with or without 0.5 or 0.75 mM$Pb(NO_3)_2$ for 2 weeks. Data are presented as the means ±SE, n = 3. Statistical significance was determined using ANOVA in combination with post-hoc (tukey) tests; significant differences ($P < 0.05$) are indicated by different lowercase letters.

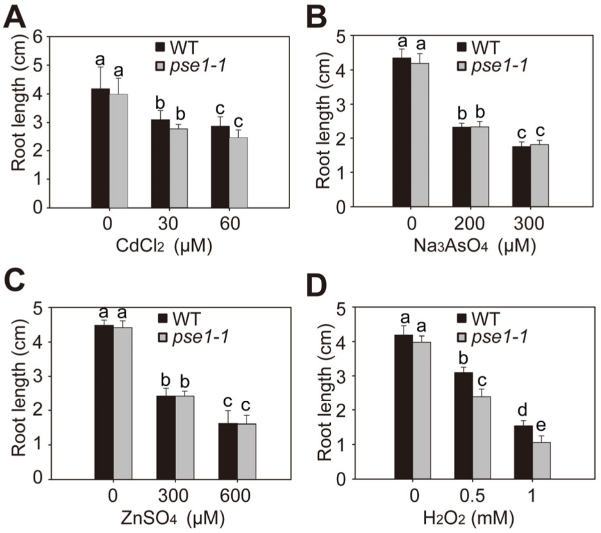

Figure S2 Phenotype of wild-type (WT) and *pse1-1* mutant seedlings in response to Cd, As, Zn, or H_2O_2 stress. The WT and *pse1-1*plants were grown on 1/2 strength MS medium with or without $CdCl_2$ (A), Na_3AsO_4 (B), $ZnSO_4$ (C), or H_2O_2 (D) for 2 weeks, and the root length and fresh weight were measured. Data are presented as the means ±SE, n = 3. Statistical significance was determined using ANOVA in combination with post-hoc (tukey) tests; significant differences ($P < 0.05$) are indicated by different lowercase letters.

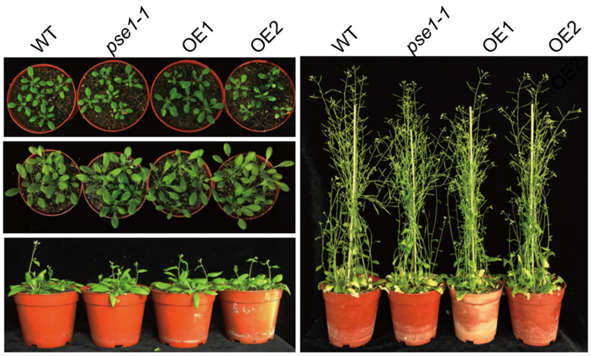

Figure S3 Phenotypes of wild-type (WT) and *pse1-1* plants in soil-filled pots.

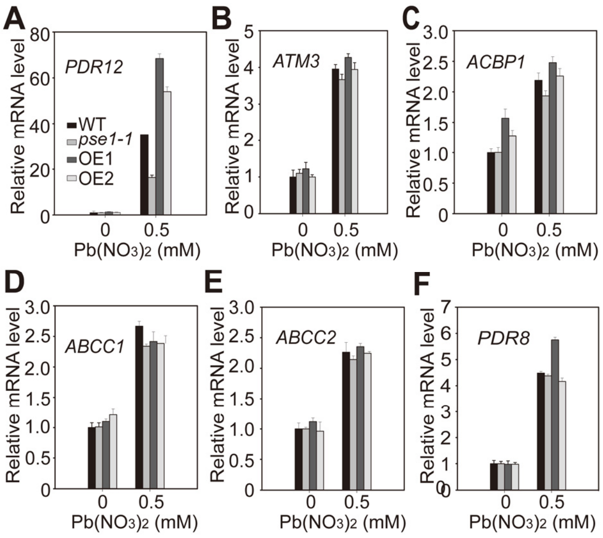

Figure S4 Transcript levels of Pb stress-related genes in WT, *pse1-1* mutant and *PSE1*-overexpressing plants subjected to Pb stress. Relative expression of *PDR12* (A), *ATM3* (B), *ACBP1* (C), *ABCC1* (D), *ABCC2* (E), and *PDR8* (F) was compared between two-week-old seedlings treated or not treated with 0.5 mMPb$(NO_3)_2$ for 12 h using qPCR. Data are presented as the means ±SE, n = 3.

Table S1 Primer sequences used for cloning and qRT-PCR

Primer Name	Primer Sequences(5'-3')
**For cloning**	
PSE1-FP-XhoI	CTCGAGATGGGTCTTCTCTCCAACAGA
PSE1-RP-HindIII	AAGCTTTCAACTCGTGCTTGGTAAAGCTATG
$PSE1_{pro}$-FP-KpnI	GGTACCATTCAGATTTTTTTTATTAACCGA
$PSE1_{pro}$-RP-XhoI	CTCGAGCTCTTCTTAGTTTCTGCAACGTCG
PSE1-GFP-FP-XhoI	CTCGAGATGGGTCTTCTCTCCAACAGA
PSE1-GFP-RP-HindIII	AAGCTTACTCGTGCTTGGTAAAGCTATG
pse1-1-LP	TGTGATCAGAGAATGGTGTGG
pse1-1-RP	CGCTTGCAACAATTACGAATC
**For qRT-PCR**	
AtPSE1-FP	AATGCGGCTTCTGACTACAA
AtPSE1-RP	GTCCTCTGCTTCAACTTTCG
AtGSH1-FP	GATGGTTTAGAGCGCAGAGG
AtGSH1-RP	TACGCTTTGTCCCCATTCTC
AtGSH2-FP	ACCAACTGCATTCCCAGAAG
AtGSH2-RP	GCCATCCAAGCTAACACGAT
AtPCS1-FP	TCAGGGATCAAAGACCAAGC
AtPCS1-RP	CCGTCGAAGATGCAATACCT
AtPCS2-FP	ATCCTCCTCACTGGGTTCCT
AtPCS2-RP	GGTTCTCTGTGGGGTCTTGA
AtGR1-FP	AGAATTTCCCAAGCGTGCTA
AtGR1-RP	AGTGCCCTCATTTCGTCATC
AtGR2-FP	TCCCAATCGCAATTCATCTAC
AtGR2-RP	TGGTAATAGCGGTGGTTTGAG
AtPDR8-FP	CTCTTGATTGGTACAGTCTTCTG
AtPDR8-RP	CCATAATGGTCCTCAATGTATTGC;
AtATM3-FP	TGCTCGGACATTTTTGAAATC

AtATM3-RP	GTCCATAGCTGCGCATATCTC
AtPDR12-FP	CAGGAAATGATAGAGCAAGGCAC
AtPDR12-RP	CGTTTTGTAATCCGAGGAAGAGA
AtABCC1-FP	GTTGACTGCGTCATTAGCCG
AtABCC1-RP	AACTGAGAAGCAAACCCATCG
AtABCC2-FP	ATTTCAGCGTGGGACAGAGG
AtABCC2-RP	ATTGAGACGGTGAGCGATA
AtACBP1-FP	AACCACACGACTCAATCGGA
AtACBP1-RP	TTTCGACACCTTCCCAATCA
ACTIN11-FP	GATTTGGCATCACACTTTCTACAATG
ACTIN11-RP	GTTCCACCACTGAGCACAATG
